# Impact of Different Stoppers on the Composition of Red and Rosé Lagrein, Schiava (Vernatsch) and Merlot Wines Stored in Bottle

**DOI:** 10.3390/molecules25184276

**Published:** 2020-09-18

**Authors:** Fabrizio Rossetti, Alicia Jouin, Michael Jourdes, Pierre-Louis Teissedre, Roberta Foligni, Edoardo Longo, Emanuele Boselli

**Affiliations:** 1 Mérieux NutriSciences, via Marradi 41, 59100 Prato, Italy; fabrizio.rossetti@mxns.com; 2 Unité de recherche Œnologie, Universitè de Bordeaux, EA 4577, USC 1366 INRA, ISVV, 210 Chemin de Leysotte, 33882 Villenave d’Ornon CEDEX, France; aliciajouin@yahoo.fr (A.J.); michael.jourdes@u-bordeaux.fr (M.J.); pierre-louis.teissedre@u-bordeaux.fr (P.-L.T.); 3 Department of Agricultural, Food and Environmental Sciences, Università Politecnica delle Marche, Via Brecce Bianche, 60100 Ancona, Italy; r.foligni@pm.univpm.it; 4 Faculty of Science and Technology, Free University of Bozen-Bolzano, Piazza Università 5, 39100 Bolzano, Italy; emanuele.boselli@unibz.it; 5 Oenolab, NOITechPark Alto Adige/Südtirol, Via A. Volta 13B, 39100 Bolzano, Italy

**Keywords:** cork stoppers, red wine, rosé, chemometrics, bottle storage, sensory analysis, two-way ANOVA

## Abstract

Different technical cork stoppers (blend of natural cork microgranules, one-piece natural cork, agglomerated natural cork, technical cork 1+1) were compared to evaluate the effects on the phenolic, volatile profiles and dissolved oxygen in three red (Merlot, Lagrein red, St. Magdalener) and one rosé (Lagrein rosé) wines, which were stored in bottles for 12 months. Gallic acid, caffeic acid, *p*-coumaric acid, *trans*-resveratrol, glutahionyl caftaric acid and protocatechuic acid did not vary remarkably during the first three months, whereas at six months a net increase was reported, followed by a clear decrease at 12 months. The same trend was observed in Lagrein rosé, but only for gallic acid. The total anthocyanins content decreased during the storage period in the four wines. Isopentyl acetate, 1-hexanol, ethyl hexanoate, 2-phenylethyl alcohol, diethyl succinate, ethyl octanoate and ethyl decanoate were the main volatile compounds. The sum of alcohols decreased in all four of the wines. The esters decreased in Merlot; however, they increased in Lagrein rosé, Lagrein red and St. Magdalener during the storage. Univariate and multivariate statistics and a sensory discriminant triangle test displayed similar influences of the different stoppers on the phenolic and volatile compounds during the whole storage duration. The changes detected in the phenolic and volatile concentrations were presumably due to the non-oxygen-mediated reactions occurring during 12 months of storage in bottle.

## 1. Introduction

Between production and consumption, wine (especially red) spends a considerable part of its life cycle stored in the bottle. Differently from most food products, which are at their best quality level when fresh, wine needs an ageing period to reach its optimum quality. In the case of red wine, the quality is expected to evolve during the storage period, which can last even decades for some premium red wines [1]. Wine is a complex matrix that undergoes several changes during bottle storage involving chemical and organoleptic properties. Color, aroma, taste and mouthfeel are related to the quality of the product [2,3]. For example, phenolic compounds are responsible for several reactions in wine during bottle storage, such as changes in color, mouthfeel and oxidation level [4,5]. The aromatic characteristics of a young wine is primarily due to fruity and floral aromas derived from fermentation. During ageing, wine aroma tends to evolve as a result of the occurrence of numerous reactions. The excess of oxygen readily induces oxidative spoilage, which results in some off-odors such as cooked vegetable, woody, liquor and animal [6,7] in the bottled wine. Other factors may also interact to affect the chemical profile, such as the light transmittance spectrum, due to the color of the bottle [8].

Moreover, oxygen can enter the bottle through the stopper at a rate dependent on the type of closure [9,10]. The parameter defining the amount of oxygen permeating a material is the oxygen transmission rate (OTR), which is often applied to assess the quality of wine closures [11]. Godden et al. [12] have confirmed the influence of the sealing system and the OTR on the evolution of wine during bottle storage. However, it may be misleading to consider the OTR as the only parameter related to the susceptibility of wine to oxidation during storage. In fact, aeration occurs in the different steps of the winemaking process starting from grape harvest and pressing [13] to the final package, and may dramatically affect the final wine quality. Determining the oxygen dissolved in wine before bottling has been the objective of extensive research [14]. While it is relatively easy to control the oxygen incorporation in tanks and vats during fermentation with a real-time approach [15,16], the non-destructive monitoring of oxygen transfer during bottle storage for the comparison of different types of stoppers still remains a challenge [17]. Several aspects should be taken into consideration: Air can enter a bottle through the interface between the stopper and the bottleneck. The stopper itself can release oxygen after the compression at bottling, a phenomenon known as outgassing [18]. Hence, understanding the influence of the stopper may help to preserve the quality characteristics of wine against undesired alteration [10].

So far, few investigations have concerned the evolution of phenolic content after bottling in relation to type of stopper, storage time [19,20] and oxygen content at bottling [21]. Other studies have outlined the relationships between the evolution of volatile profile and aroma in bottle-aged wines while considering oxygen content [22] and the use of different types of stoppers in Cabernet Sauvignon [23].

In this study, both the phenolic and volatile profiles of three red and one rosé wines from South Tyrol (Northern Italy) were monitored regularly over 12 months of bottle storage. The aim of this research was to investigate the influence of different types of stoppers on the evolution of wine quality, including a sensory evaluation. The outcomes of this study will provide new applied knowledge on stopper–wine interaction phenomena, as well as guidelines for the preservation of the quality attributes during the bottle storage of wines obtained from specific red varieties.

## 2. Results

### 2.1. Evolution of Phenolic Compounds

The profile of non-anthocyanin polyphenols is reported in Table 1.

Ten non-anthocyanin phenolic compounds were identified. Although each of the four wines had a different phenolic profile due to the different composition of the grape varieties used for winemaking, a common evolution trend was detected for six out of 10 compounds, namely, gallic acid, caffeic acid, *p*-coumaric acid, *trans*-resveratrol, glutathionyl caftaric acid (GRP) and protocatechuic acid. During the first three months of bottle storage (T1, T2, T3) their concentrations did not show notable variations. At six months (T4), a net increase was observed, followed by a clear decrease at 12 months (T5). This trend was detected in the three red wines; however, in Lagrein rosé, this evolution was registered only for gallic acid, but not for the other phenolic compounds. A decrease of the low molecular weight compounds such as caffeic acid, (+)-catechin, (−)-epicatechin and *p*-coumaric was observed when comparing the final concentrations (T5) with the initial ones (T1), as already reported in previous studies [19,24].

The anthocyanins were derivatives of delphinidin, cyanidin, petunidin, peonidin and malvidin, which are the typical anthocyanins present in wines obtained from *Vitis vinifera* L. red grape varieties. Anthocyanins were grouped in three families according to the esterification groups: glucosides, acetyl-glucosides and coumaroyl-glucosides. Their concentrations are reported in Table 2.

The glucosides-anthocyanins included delphinidin-3-*O*-glucoside, cyanidin-3-*O*-glucoside, petunidin-3-*O*-glucoside, peonidin-3-*O*-glucoside and malvidin-3-*O*-glucoside. Acetyl-glucosides included delphinidin-3-*O*-acetylglucoside, petunidin-3-*O*-acetylglucoside, peonidin-3-*O*-acetylglucoside and malvidin-3-*O*-acetylglucoside. Coumaroyl-glucosides grouped petunidin-3-*O*-coumaroylglucoside, peonidin-3-*O*-coumaroylglucoside and malvidin-3-*O*-coumaroylglucoside. At bottling time T1, the Lagrein red wine had the highest concentration of anthocyanins compared to Merlot and St. Magdalener. The total anthocyanins content of Lagrein was almost double that of Merlot, whereas non-acylated anthocyanins in Lagrein were about 265 mg L^−1^ compared to about 160 mg L^−1^ for Merlot and about 142 mg L^−1^ in St. Magdalener. Acetylated and coumaroylated anthocyanins were also present in higher concentrations in Lagrein red (86 mg L^−1^ and 27 mg L^−1^, respectively), followed by Merlot (48 and 19 mg L^−1^, respectively) and St. Magdalener (29 and 13 mg L^−1^, respectively). Rosé wine produced from Lagrein had a predictably much lower concentration of anthocyanins (non-acylated, about 28 mg L^−1^; acetylated, about 14 mg L^−1^; coumaroylated about 7 mg L^−1^) compared to the three red wines, due to the shorter maceration time. Nonetheless, in all four wines the sum of the three classes and thus the total amount of identified anthocyanins showed a clear decrease over the storage period. The reduction of the phenolic content during the bottle storage is generally ascribed to polymerization, oxidation and complexation reactions [25,26,27]. In this case the dissolved oxygen present in the four wines (Table 3) was low already just after bottling (0.15–1.7 mg L^−1^ in red and 1.4–3.1 mg L^−1^ in rosé wine) and varied slightly during 12 months of storage.

### 2.2. Effect of the Type of Stopper and Storage Time on the Phenolic Profile

Two-way ANOVA was performed to assess the influence of the type of stopper and the bottle storage time on the phenolic composition of the four wines. The results showed that the storage time (F storage time) significantly influenced the phenolic concentration (Table 1 and Table 2). Notably, GRP and anthocyanin glucosides, acetyl-glucosides and coumaroyl-glucosides in Lagrein rosé (F = 8570; 34,170; 20,423 and 10,927, respectively); caffeic acid, GRP and glucoside in Merlot (F = 3435; 3263 and 3435, respectively); GRP, glucosides and acetyl-glucosides in St. Magdalener (F = 2861; 4767 and 2971, respectively); and *p*-coumaric acid in Lagrein (F = 4297) were strongly affected by the storage period. On the other hand, the influence of the type of stopper at each sampling time (F stopper) a significantly affected the phenolic compounds only in a few cases.

Compared to the bottles closed with conventional stoppers, Lagrein red wine closed with the ‘blend’ stopper showed significantly lower concentrations of anthocyanin glucosides at three months (T3), acetyl glucosides at six months (T4), (+)-catechin and caffeic acid at three and six months and *p*-coumaric acid at one month of storage (T2). Merlot wines reported significant differences for anthocyanin acetyl-glucosides at bottling (T1), at one month (T2) and at three months (T3), but in this case higher concentrations were detected in the bottles closed with the ‘blend’ stoppers compared to the conventional stoppers. All these differences can be attributable to an imperfect homogeneity of the Merlot wine in the bottling line (for each sampling point, the content of different bottles were analyzed).

The Lagrein rosé wine showed a higher abundance of *p*-coumaric acid at T2 in the wine sample closed with the ‘blend’ stopper. St. Magdalener wines differed for the lower concentrations of GRP, anthocyanin glucosides and acetyl-glucosides after six months storage (T4) in bottles closed with the ‘blend’ stoppers.

To summarize, a common temporal trend of one or more phenolic compounds related to the type of stopper could not be evidenced in the four wines. The statistical differences highlighted between two types of stoppers were quite unpredictable and seemed to be due to the natural variability of the products.

**Figure 1:**
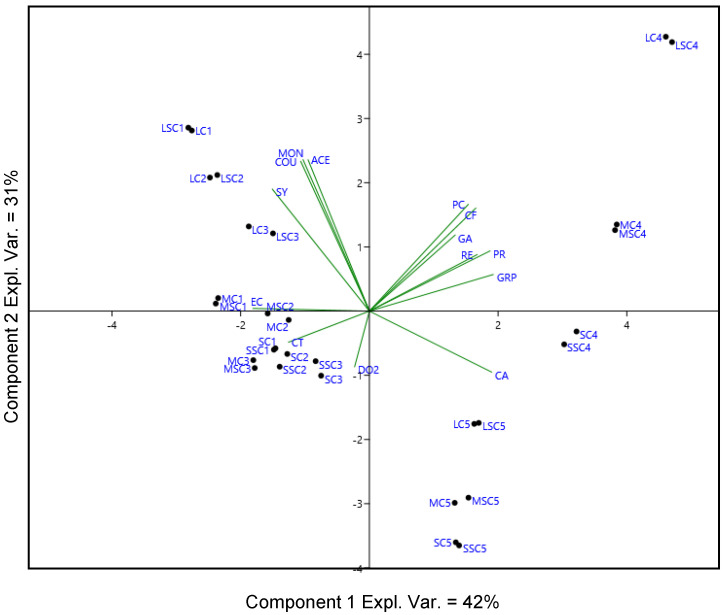
PCA bi-plot (% explained total variance: PC-1, 42%; PC-2, 31%) of the three red wines closed with different stoppers considering the phenolic profiles and the dissolved oxygen (DO_2_) during 12 months of bottle storage. Samples: SC and SSC, St. Magdalener closed with conventional stopper or ‘blend’ stopper, respectively. LC, LSC, Lagrein with conventional or ’blend’ stopper, respectively; MC, MSC, Merlot. Storage in bottle: 1, wine just after bottling; 2,3,4,5, wine analyzed one, three, six and 12 months after bottling, respectively. Phenolic compounds: MON, non-acylated anthocyanins; ACE, acetylated anthocyanins; COU, coumaroylated anthocyanins; GA, gallic acid; PR, protocatechuic acid; GRP, glutathionyl caftaric acid; CA, caftaric acid; CF, caffeic acid; CT, catechin; EC, epicatechin; SY, syringic acid; PC, *p*-coumaric acid; RE, resveratrol.

To better understand the influence of storage time and type of stopper on the phenolic profile in relation to dissolved oxygen, PCA was performed with all the phenolic compounds and the dissolved oxygen values obtained at each step of analysis. Rosé wines were excluded from the PCA because their content of phenolic compounds was lower than the red wines. The PCA plot showing the loading of variables and the scores of the samples in the bi-dimensional space is reported in Figure 1.

The first two principal components made up 73% of the model (PC-1 42%, PC-2 31%) when taken together. Samples collected at bottling time (T1), after one month (T2) and after three months (T3) were grouped on the negative side of the PC-1 axis, whereas the wines stored for six months (T4) and 12 months (T5) were placed on the positive side of the PC-1 axis. Considering the loadings, the three classes of phenolic anthocyanins (glucosides, acetyl-glucosides and coumaroyl-glucosides) and non-anthocyanin phenolic compounds, such as syringic acid, showed a positive correlation with Lagrein red samples collected at bottling time (T1), three months (T2) and at six months (T3). On the other hand, catechin and epicatechin showed a good correlation with Santa Madgalener and Merlot samples analyzed at T1, T2 and T3. Gallic acid, protocatechuic acid, GRP, caffeic acid, *p*-coumaric and trans-resveratrol were positively correlated with the Merlot and Lagrein samples collected after six months (T4). Caftaric acid characterized all the wines stored for 12 months (T5), as well as the St. Magdalener wines sampled at six months storage (T4).

The dissolved oxygen was not strongly correlated with any specific sample.

This PCA model successfully scattered the samples depending on the storage period, whereas the bottles closed with similar stoppers were not clearly separated in any case. The multivariate approach confirmed the ANOVA results, remarking the dominant influence of the storage time over the type of stopper.

### 2.3. Evolution of Volatile Compounds during the Storage in Bottle

A total number of 26 volatile compounds were identified in all the wines (Table 4).

The main goal of the SPME-GC/MS analysis was not to obtain the absolute concentrations of the volatile compounds present in the wine headspace, but to study the relationships between their relative concentrations (profiles) and the technological variables (wine type/stopper type/storage time) with a multivariate statistical approach.

The abundances of the volatile compounds (averages and standard deviations of two replicate bottles) were expressed as relative percentage (internal area) considering all the volatile compounds, as reported in Supplementary Materials (tables of volatile compounds’ average peak areas in the studied wines).

The most represented compounds were short- and medium-chain esters, which included 17 compounds, followed by higher alcohols, short- and medium-chain organic acids and terpenes. During the bottle storage, more than 80% of the total abundance was represented by seven compounds: isopentyl acetate, 1-hexanol, ethyl hexanoate, 2-phenylethyl alcohol, diethyl succinate, ethyl octanoate and ethyl decanoate. Over 12 months, the modification of the volatile profile was primarily due to the evolution of these compounds. Ethyl octanoate and ethyl decanoate showed a decreasing trend in the early period of bottle storage (T2–T4), followed by an increase until the 12th month (T5) in red wines. Isopentyl acetate increased after one month (T2) of bottle storage in the three red wines and reached its maximum abundance at three months (T3), probably as a consequence of acid-catalyzed reactions involving fatty acid esters and resulting in the production of acetate esters [38,39].

The sum of higher alcohols decreased in all four of the wines. This reduction was detected in previous studies [40,41,42] and can be explained as the result of the previously cited acid-catalyzed esterification reaction [43]. Concerning the total amount of esters, Merlot is notable for having experienced a slight reduction during the 12 months in wine closed with a conventional stopper, whereas the Merlot, Lagrein rosé, Lagrein red and St. Magdalener wines closed with the ‘blend’ stopper showed a higher total ester content at the end of the storage compared to the start of the storage. This latter trend can occur as a consequence of the esterification of branched acids to form ethyl esters [44,45,46].

**Figure 2:**
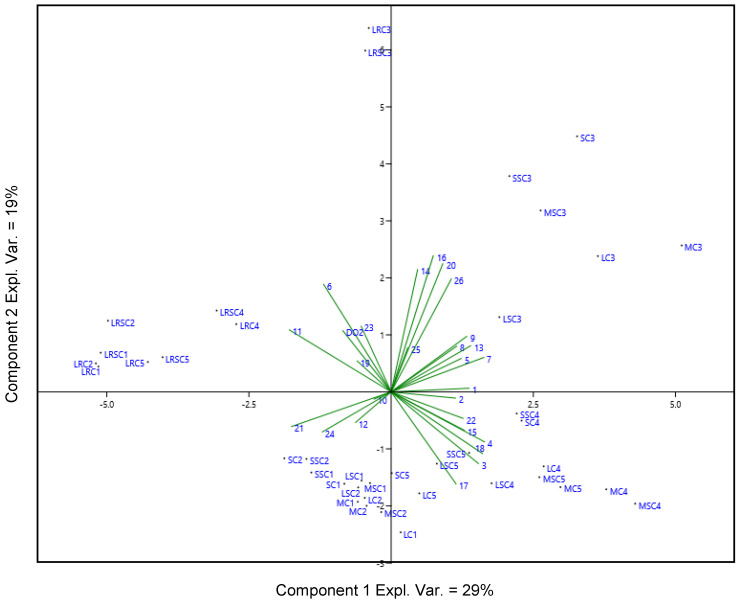
PCA bi-plot (PC-1 29%, PC-2 19%) of the three red wines and the rosé wine closed with different stoppers considering the volatile profiles and the dissolved oxygen (DO_2_) during 12 months of bottle storage. Samples: LRC, LRSC, Lagrein rosé closed with conventional or ’blend’ stopper, respectively. SC and SSC, St. Magdalener with conventional or ‘blend’ stopper. LC, LSC, same for Lagrein. MC, MSC, Merlot. Storage in bottle: **1**, wine just after bottling; **2**,**3**,**4**,**5**, wine analyzed one, three, six and 12 months after bottling, respectively. Volatile compounds are named **1**–**26** as listed in Table 4.

In order to assess further relations between the type of stopper and the volatile compounds, PCA was applied to all of the red and rosé wine samples, the volatile compounds and the dissolved oxygen levels detected during the storage period. The loadings and variables of the PCA are displayed in Figure 2.

The first two principal components explained 48% of the model (PC1, 29%, PC2, 19%). The PCA showed a separation of the rosé samples in the left part of the bi-plot with the T3 samples clustered away from all other samples along PC1. The red wines collected at bottling (T1) and after one month (T2) were grouped in the middle of the bi-plot, whereas samples stored for three months (T3) were segregated in the upper-right quadrant and samples collected after six months (T4) and 12 months (T5) were grouped together in the lower-right quadrant.

Some of the volatile compounds related to fresh fruity and floral notes, such as limonene (**12**), ethyl octanoate (**21**) and ethyl decanoate (**24**) positively correlated with samples stored for short periods (T1 and T2). On the other hand, a volatile compound connected with oxidation reaction such as diethyl succinate (**18**) distinguished samples stored for longer periods (T4 and T5). The rosé wine showed a positive correlation with 2-phenethyl acetate (**23**) and hexyl acetate (**11**); acetate esters are sensory descriptors of the fruity character of rosé wines [47]. Also, this PCA scattered the samples depending on the storage period, whereas there was a lack of differentiation among the bottles closed with the two stoppers. This explains the similar influence of the different stoppers on the volatile profiles and on the dissolved oxygen level during 12 months of storage.

No consistent significant difference in terms of stopper or time-stopper interaction was evidenced by two-way ANOVA for any wine in terms of specific variables, although some compounds showed differences at specific times for stoppers. The main significance was shown in relation to time evolution, which involved most of the volatile compounds for all wines, as shown for the phenolic compounds. These will be discussed in more details in relation to the sensory test results.

### 2.4. Hierarchical Clustering Analysis (HCA)

**Figure 3:**
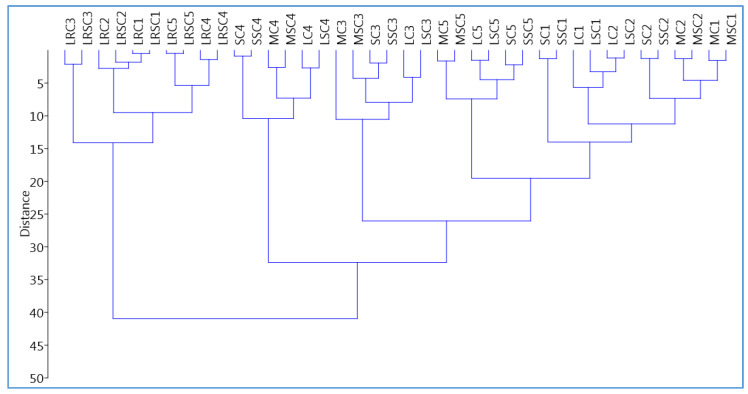
Hierarchical cluster analysis (HCA) of the four wines during 12 months of bottle storage. Samples: LRC, LRSC, Lagrein rosé closed with conventional or ’blend’ stopper, respectively. SC and SSC, St. Magdalener with conventional or ‘blend’ stopper. LC, LSC, same for Lagrein. MC, MSC, Merlot. Storage in bottle: **1**, wine just after bottling; **2**,**3**,**4**,**5**, wine analyzed one, three, six and 12 months after bottling, respectively.

HCA was performed to assess the overall similarities and differences between the wines using the phenolic and volatile compounds identified as variables. The dendrogram clearly segregated the Lagrein rosé wine in a single branch of the diagram, separated from the three red wines (Figure 3).

The Merlot, Lagrein red and St. Magdalener wines were clustered at a close hierarchical distance at T1 and T2, highlighting similarities to each other. Samples obtained after three and 12 months after bottling (T3 and T5) were segregated in two adjacent branches, whereas samples after six months bottling were isolated in an individual branch. This result shows that the evolution of the wines started after three months (T3), and reached the maximum differentiation after six months (T4). At 12 months of bottle storage, the composition of the wines changed again and became more similar to its initial state. Regarding the type of stopper, with HCA the wine bottles closed using the two conventional stoppers were at a close hierarchical distance, thus explaining the similar influence of similar stoppers on the phenolic and volatile profiles.

### 2.5. Sensory Analysis

The results of the discriminant triangle test (Table 5) showed significant differences for St. Magdalener and Merlot wines closed with the two different stoppers at the bottling time (T1), probably due to imperfect homogeneity of the wine in the bottling line. A significant number of assessors also recognized differences concerning Merlot wine in the tasting session performed after six months of storage (T4). Conversely, the required number of correct answers for differentiating the two samples closed with the different stoppers for any of the four wines was not obtained in the other tasting evaluations. An overall view of the tasting sessions highlights only few cases of significant differences. So, the differences were presumably due to the natural variability of the product.

**Figure 4:**
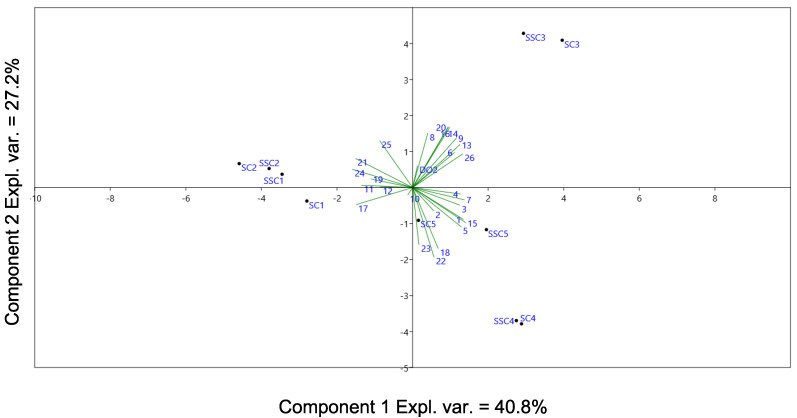
PC1 (exp. variance = 40.8%) vs. PC2 (exp. variance = 27.2%) model for St. Magdalener wines. Volatile compound relative abundances and measured dissolved oxygen were used as variables. Volatile compound labels (**1**–**26**) are explained in Table 4.

Further investigations were performed to identify the compounds possibly responsible for the correct discrimination of St. Magdalener at T1 and Merlot wine at T1 and T4. A PCA model was built for St. Magdalener samples upon volatile compounds in order to identify the species most relevant for for the differentiation of the samples (Figure 4).

The largest separation was identified between three groups of samples: T1-T2, T3 and T4, with T5 being more or less precisely at the center of the PCA model (considering PC1 and PC2). Two-way ANOVA was applied on all compounds to study the significance (*p*-value < 0.05) of the differences across the samples for specific variables, in particular with respect to the time evolution between stoppers and their interaction. Significant differences for the stopper were found for 2-ethylhexanol (13, *p*-value ~ 0.05), 1-octanol (15, *p*-value ~ 0.05). Significant differences for time evolution were found for acetic acid (**1**), 1-hexanol (**5**), isopentyl acetate (**6**), 2-butyl 4-ethylbenzoate (**7**), 1-heptanol (**8**), 1-octen-3-ol (**9**), hexyl acetate (**11**), limonene (**12**), 2-ethylhexanol (**13**), methyl benzaldehyde (**14**), 1-octanol (**15**), ethyl benzaldehyde (**16**), 2-phenylethanol (**17**), diethyl succinate (**18**), octanoic acid (**19**), methyl salicylate (**20**), ethyl octanoate (**21**), ethyl benzeneacetate (**22**), 2-phenyl acetate (**23**), ethyl decanoate (**24**), ethyl dodecanoate (**25**) and ethyl hexadecanoate (**26**). No significant interaction between the two factors was found.

Only borderline differences (at *p*-value ~ 0.05) were observed for the type of stopper. ANOVA indicated that the only significant differences observed between the samples were occurring as a time evolution, and in agreement with the interpretation of PC1 and PC2 in Figure 4. In fact, no variable was able to differentiate wines for the stoppers at a specific time.

**Figure 5:**
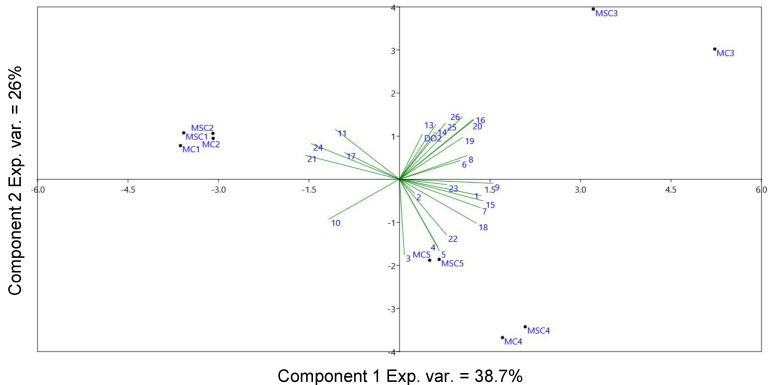
PC1 (exp. variance = 38.7%) vs. PC2 (exp. variance = 26%) model for Merlot wines. Volatile compound relative abundances and measured dissolved oxygen were used as variables. Volatile compounds labels (**1**–**26**) are explained in Table 4.

As done with St. Magdalener, a PCA model was built on Merlot samples upon volatile compounds in order to identify the volatiles most differentiating the wines by the stopper (Figure 5).

T1 and T2 samples were tightly clustered at negative values of PC1 and PC2. T3 samples were found at the highest positive values of PC1 and PC2, while at the same time completely separate from all other samples. T4 samples were found at the most negative values of PC2, whereas T5 samples were grouped in the same general direction, although much closer to the center of the PCA model with respect to both principal components. T4 samples showed again (as for St. Magdalener) to be between T3 and T5 with respect to PC1; similarly, the evolution from T3 to T5 appeared to opposed that from T1 to T3 along PC1. The separation of T4 from T3 and T5 occurred along PC2, but T3 samples were farther away than T5 in that direction, therefore indicating that these T4 samples could be regarded as a turning point in the wine evolution, with respect to the variables most relevant for PC2. Two-way ANOVA was applied to investigate the significance (*p*-value < 0.05) of differences for the time evolution, stoppers and possible factor interactions. As a result, significant differences (s.d.) were found with respect to the stopper for ethyl butanoate (2; *p-*value ~ 0.05), methyl benzaldehyde (**14**), 1-octanol (**15**), octanoic acid (**19**) and methyl salicylate (**20**).

Significant differences with respect to time were found for ethyl 2-methylbutanoate (**3**), ethyl 3-methylbutanoate (**4**), 1-hexanol (**5**), isopentyl acetate (**6**), 2-butyl 4-ethylbenzoate (**7**), 1-heptanol (**8**), ethyl hexanoate (**10**), 2-ethylhexanol (**13**), 4-methylbenzaldehyde (**14**), 1-octanol (**15**), 4-ethylbenzaldehyde (**16**), diethyl succinate (**18**), octanoic acid (**19**), methyl salicylate (**20**), ethyl octanoate (**21**), ethyl benzeneacetate (**22**), ethyl decanoate (**24**), ethyl dodecanoate (**25**) and ethyl tetradecanoate (**26**). Significant interactions between time and stopper were found for ethyl butanoate (**2**), 4-methylbenzaldehyde (**14**), 1-octanol (**15**), octanoic acid (**19**) and methyl salicylate (**20**). These were the same compounds differentiating the wines for stoppers. A Tukey’s HSD test highlighted how several differences were found for time between the two stoppers (hence the interaction), but also that all these compounds differentiated the wines at T3 between the two stoppers. The main differences were seen overtime. In particular, no specific compound could be indicated as responsible for the difference between the two stoppers at T4. Further, as seen in Table 1 and Table 2 and Section 3, the phenolic compounds’ profiles (anthocyanins and non-anthocyanins) did not account for the differences observed in the triangle tests; instead, they mostly showed a dependence on storage time in the PCA (Figure 1), with the largest differences at T3 for all wines, mostly due to storage time, with few exceptions. Therefore, further investigations (especially in terms of descriptive sensory analysis and quantitative sensory models) will be therefore required in order to be correlated to the parallelly studied chemical profiles.

## 3. Discussion

This work explores the influence of type of stopper and storage period on the phenolic and volatile profiles of less-studied red and rosé wines from Lagrein and Schiava/Vernatsch red grape varieties during 12 months of bottle storage. Further information on the evolution of the phenolic and volatile compounds in these three red and one rosé wines from Northern Italy has been provided.

The total phenolic content clearly decreased during the bottle storage, with a remarkable loss recorded between six and 12 months, as observed in previous studies on a Cabernet Sauvignon [18] and at 10 months of bottle storage in a rosé wine from Grenache grapes [4]. Epicatechin, non-acetylated anthocyanins, acetylated anthocyanins and coumaroylated anthocyanins were those compounds showing the highest inverse correlation with the change from T1 to T5 (i.e., decreasing over time), and thus the most likely to undergo oligomerization reactions. The non-oxygen mediated nature of this transformation is also supported by the fact that the variation in the amount of dissolved oxygen was completely not correlated with the evolution of these variables from T1 to T5 in the PCA model built on the phenolic compounds.

Over 12 months of storage, changes in the volatile profiles resulted in lower concentrations of higher alcohols in all the four wines, as reported in previous studies [40,41,42]. Concerning the total amount of esters, Lagrein rosé, Lagrein red and St. Magdalener wines closed with the ‘blend’ stopper showed a higher abundance at the end of the storage compared to the beginning. A similar increasing trend was reported for the majority of ester compounds in Cabernet Sauvignon stored in bottles for nine months [23].

In conclusion, the present study shows that the type of stopper did not consistently influence the chemical profile evolution of the four wines. Only in few cases was a correlation observed in the evolution of the volatile compounds, although not in a consistent way. This study shows instead the time evolution of the four wines. Considering the low levels of dissolved oxygen, the changes detected in the phenolic and volatile concentrations were presumably due to non-oxygen-mediated reactions occurring during the 12 months of bottle storage, and in fact for the volatile profile they mainly involved ester formations.

Time evolutions were observed for all wines and involved most of the chemical variables identified. These results agree with previous studies outlining no significant differences due to type of stopper on the evolution of chemical and sensory profiles [20]. However, this observation may still be related to the type of wine studied, as some exceptions are present in previous works for synthetic, screw-cap and glass closures vs. natural cork closures for Chardonnay wines [2]. As recently reviewed [18], control over wine exposure to oxygen during and after bottling operations is required to positively influence the desired evolution of chemical and sensory profiles, although these will strongly evolve depending on the type of wine. Further studies may consider drawing a model that more closely relates the sensory and chemical profiles, as well as extending the investigation beyond 12 months to assess major influences of the different stoppers on the evolutions of phenolic and volatile profiles.

## 4. Materials and Methods

### 4.1. Wine Samples and Stoppers

Four Italian wines were provided by a winery located in South Tyrol (Kellerei Bozen, Bolzano, Italy). Merlot and Lagrein red and Lagrein rosé wines were obtained only from the corresponding local grapes (monovarietal wines), whereas the St. Magdalener wine was a blend obtained from Schiava (dominant) and Lagrein grape varieties. All the grapes were harvested in 2016 from vineyards located in the South Tyrol area, and the wines were bottled within the following year. The winery provided a total number of 80 bottles (0.75 L)—20 bottles for each type of wine. The wine bottles were sealed with different stoppers, and a stopper consisting of a sanitized cork micro-granule blend of natural cork with polymers without the addition of glue to the mix (‘blend’ stopper, Supercap srl, Mombaroccio, Italy) was compared with three different types of stopper (one-piece natural cork, agglomerated natural cork, technical cork 1+1) according to the scheme outlined in Table 6. The bottles were stored horizontally and protected from light, first at a cellar temperature, then in a laboratory with constant controlled temperature (23 °C) and medium relative humidity. The analyses were performed at five specific time points: at bottling (T1), after one month (T2), at three months (T3), at six months (T4) and at 12 months (T5) of storage at cellar temperature. At each sampling time, four bottles of each wine (two bottles closed with the same stopper, thus two replicates for each stopper type) were analyzed. 

### 4.2. Chemicals and Reagents

All reagents and chemical standards used in this study were purchased from Sigma-Aldrich S.p.A. (Milan, Italy), whereas the solvents (analytical grade) were obtained from VWR International s.r.l. (Milan, Italy).

### 4.3. Non-Anthocyanin Phenolic Compounds

The analysis of the non-anthocyanin polyphenols (phenolic acids, flavan-3-ols, stilbenes) was performed through a UHPLC-DAD-HRMS system (Agilent 1290 Infinity, Les Ulis, France) equipped with a UV-Vis diode array detector (DAD) (1290 Infinity) connected to the quadrupole time of a flight mass spectrometer (QToF/MS) with an electrospray ionization (ESI) source (Agilent 6530 Agilent Accurate Mass). The UHPLC-DAD-HRMS analyses were carried out on a C18 reversed-phase column (2.1 × 100 mm, 1.8 µm, Agilent). Samples were directly filtered on a 0.45-µm-membrane filter before the injection. The temperature of the column was 25 °C and the flow rate was set to 0.3 mL/min, with the injection volume set at 2.0 µL. The mobile phases consisted of (A) aqueous 0.1% formic acid and (B) methanol with an addition of 0.1% formic acid. The gradient of solvent B was as follows: 6% for 0.5 min, 6% to 40% for 29.5 min, 40% to 100% for 8 min, 100% for 5 min and back to 6% in 2 min, followed by washing and re-equilibration for 3 min. The mass spectrometer was operated in the extended dynamic range of 2 GHz (*m*/*z* 3200). The nebulizer pressure and flow rate were set at 25 psi and 9 L min^−1^, respectively. Its drying gas temperature was 300 °C. The sheath gas flow and temperature were set at 11 L min^−1^ and 350 °C. The fragmentation, skimmer, OCT and capillary voltage were set at 150, 65, 750 and 4000 V, respectively. The analyses were performed in negative ionization mode. The data analysis was performed on Mass Hunter Qualitative Analysis software (version B.06.00). Phenolic compounds were identified by comparing their chromatographic retention times and accurate masses with those of pure standard compounds. The calibration curves of pure standard substances were established through the DAD and were used to quantify the phenolic concentrations. When reference compounds were not available, a calibration with structurally related standard substances was used (gallic acid for protocatechuic acid and syringic acid; caffeic acid for caftaric acid and glutathionyl caftaric acid (GRP); (+)-catechin for (−)-epicatechin). The integrated peaks were allowed to obtain the concentrations of the identified compounds. Concentrations were expressed in mg L^−1^ of standard or of the structurally related standard.

### 4.4. Anthocyanins

The relative composition of three anthocyanins classes (glucosides, acetyl-glucosides and coumaroyl-glucosides) was determined using a method slightly modified from [48]. Samples were directly filtered on a 0.45-µm-membrane filter before analysis. The HPLC system (Accela series, Thermo-Scientific, Illkirch-Graffenstaden, France) was equipped with a 4 × 250 mm internal diameter (i.d.), 5-µm Nucleosil C18 column (Agilent). The solvents used were water (Eluent A) and acetonitrile (Eluent B), both containing 5% formic acid. The gradient of solvent B consisted of 10%–23% in 16 min, 23%–28% in 19 min and 28%–100% in 6 min at a flow rate of 1 mL/min. The column was washed with 100% acetonitrile for 5 min and re-equilibrated with the initial conditions for 3 min [49]. The tentative identification of the analytes was performed in accordance with bibliographic data. The quantification was carried out through the injection of malvidin-3-*O*-glucoside (Mv3G) as an external standard.

### 4.5. Volatile Compounds

The profile of the volatile compounds was obtained by gas-chromatography mass-spectrometry (GC-MS) after extraction with head-space solid-phase micro-extraction (HS-SPME) according to a published procedure [50], with slight modifications. Briefly, 10 mL of wine were introduced into a 20-mL vial and 1 g NaCl was added. After mixing, the vial was tightly capped with a screw cap equipped with a perforable elastomeric septum. The vial was equilibrated in a heating bath at 40 °C for 10 min. Afterwards, a SPME fiber coated with 50/30 µm divinylbenzene/carboxen/polydimethylsiloxane (DVB/CAR/PDMS; Supelco/Sigma-Aldrich, Milan, Italy) was inserted into the vial and exposed to the sample headspace for 20 min under continuous heating. Subsequently, the thermal desorption took place in the GC injector at 220 °C for 3 min. A Varian 3900 gas-chromatograph coupled to a Saturn 2100T (Varian, Walnut Creek, CA, USA) ion trap mass spectrometer was equipped with a ZB-5 capillary column (Phenomenex, 30 m × 0.25 mm I.D., film thickness 0.25 µm). The injection was in splitless mode (splitless time 0.3 min) and the temperature program of the GC oven was conducted as follows: holding at 40 °C for 10 min, then raising up to 180 °C at a rate of 3 °C min^−1^ before reaching 250 °C at 15 °C min^−1^. The MS transfer line and trap temperatures were set at 200 °C. The ion trap emission current was 10 µA. The mass spectra were recorded in the full scan mode (mass range 31–250 *m*/*z*) at 1 scan s^−1^. Data were analyzed with the Varian Workstation software. Tentative identification was based on the comparison with the NIST library mass spectra (Version: 2.0; 2002), the GC linear retention indices reported in the literature and through the injection of pure standard substances [51], when available. Samples were analyzed in duplicate (two different bottles for each stopper type). Quantification of the peaks area was expressed as an internal area percentage.

### 4.6. Dissolved Oxygen Content

The dissolved oxygen content of the wines was measured using a non-invasive optical L.sensor-700.O_2_ (FT system, Alseno, Italy). The technology used an IR laser type; the range of the measurement was between 0.3% and 21% O_2_ ± 0.3%; the accuracy was ±0.2% (oxygen concentration).

### 4.7. Sensory Analysis

A sensory panel (10 women and eight men, ages 23–60 years) was formed by professional wine judges from the enology research unit of the Institut de Science de la Vigne et du Vin (ISVV, Bordeaux, France). The sensory sessions were conducted in a tasting room provided with individual booths according to the ISO 8589 at the same research unit (ISVV).

The discriminant triangle test (ISO 4120: 2007) was chosen to evaluate the possible differences between each of the four wines closed with a conventional cork stopper and compared to the ‘blend’ stopper at each storage time. The sensitivity parameters of the test were set at α = 0.5, β = 0.20 and *p*_d_ = 50%.

The judges received four sets of three wines and were asked to evaluate each set per time selecting the odd sample of each set. The glasses were labeled with three-digit random codes and were presented to the panelists according to a random order, as prescribed by the standard methodology for the triangle test in sensory analysis (ISO 4120:2007). Each session was one day after the first.

### 4.8. Statistical Analysis

All the chemical data refer to the determination of two replicate bottles for each type of stopper, and each of the four types of wine were sampled at each of the five time intervals (T1–T5). For the volatile compounds, data are reported as internal area averages of the two replicates with the related standard deviation. Two-way analysis of variance (two-way ANOVA) was performed using GraphPad Prism v6.01 software (San Diego, CA, USA) with the storage time and stopper type as independent variables. When significant differences were revealed (*p* < 0.05), a Tukey’s (HSD) multiple comparison test was applied to compare the mean concentrations.

A Student’s *t*-test was applied to compare the dissolved oxygen concentrations into the wines closed with the comparative stoppers (*p* < 0.05).

Multivariate statistics was applied to the normalized data of the phenolic and volatile profiles. Hierarchical cluster analysis (HCA) was performed to assess the similarities among the wines employing Euclidean distance and Ward’s linkage method. Principal component analysis (PCA) was carried out to evaluate the influence of phenols and volatiles on the wines closed with the different stoppers. PCA and HCA were both performed using PAST software v3.18 [52]. The sensory data obtained from the triangle test sessions were analyzed according to the ISO 4120—Sensory analysis—Methodology.

## Figures and Tables

**Table 1:** Non-anthocyanin phenolic compounds (mg L^−1^) and significance of the type of stopper and storage time on their modifications.

	Gallic Acid						Protocatechuic Acid					Caftaric Acid					GRP					(+)-Catechin				
	Time	c	Blend	*F1*	*F2*	*F3*	c	Blend	*F1*	*F2*	*F3*	c	Blend	*F1*	*F2*	*F3*	c	Blend	*F1*	*F2*	*F3*	c	Blend	*F1*	*F2*	*F3*
Lagrein Red	T1	16.2 ± 0.5	16.3 ± 0.1	ns	1932 *	ns	1.4 ± 0	1.3 ± 0.1	ns	1143 *	ns	19.9 ± 1.2	18.8 ± 0.7	ns	356.5 *	ns	1 ± 0.1	1 ± 0.1	ns	626.7 *	ns	23.7 ± 0.1	24.2 ± 2.5	14.1 *	81.7 *	ns
	T2	17 ± 0	18 ± 0.5			ns	1.2 ± 0.1	0.9 ± 0			ns	20.3 ± 0.2	23.7 ± 1.2			ns	1 ± 0.1	0.8 ± 0			ns	24.9 ± 1.2	24.6 ± 2.2			ns
	T3	18.7 ± 1.4	18.4 ± 0.1			ns	1.4 ± 0	2.2 ± 0.7			ns	22.1 ± 0.1	20.6 ± 0.8			ns	1 ± 0	1 ± 0.1			ns	23.9 ± 0.3	18 ± 0.4			*
	T4	47.6 ± 0.7	46.5 ± 0.1			ns	9.9 ± 0.1	10.3 ± 0.3			ns	41.5 ± 3.5	38.8 ± 0			ns	17.3 ± 1.8	16.3 ± 0.4			ns	20 ± 0.2	12.5 ± 0.5			*
	T5	21.7 ± 0.2	21.4 ± 0.4			ns	3.3 ± 0.1	3.5 ± 0.1			ns	47.5 ± 0.1	47.2 ± 0			ns	1.5 ± 0.1	1.5 ± 0.1			ns	15.7 ± 0.2	14.7 ± 0.5			ns
Lagrein Rosè	T1	3.8 ± 0.1	4.1 ± 0.1	ns	751 *	ns	0.5 ± 0	0.5 ± 0	ns	27 *	ns	3.8 ± 0	3.8 ± 0.2	ns	786 *	ns	1.4 ± 0	1.3 ± 0.1	ns	8570 *	ns	7.1 ± 0.2	7.4 ± 0.6	ns	29 *	ns
	T2	3.7 ± 0.2	4 ± 0.2			ns	0.4 ± 0	0.4 ± 0			ns	4.2 ± 0.2	4.5 ± 0.1			ns	1.3 ± 0.1	1.3 ± 0.1			ns	5.4 ± 0.4	5.6 ± 0.2			ns
	T3	4.6 ± 0.1	4.9 ± 0			ns	0.6 ± 0.1	0.5 ± 0			ns	3.7 ± 0.1	3.8 ± 0.1			ns	1.2 ± 0	1.2 ± 0			ns	5 ± 1.1	5 ± 0			ns
	T4	6 ± 0.1	5.9 ± 0.1			ns	0.6 ± 0.1	0.5 ± 0			ns	6.8 ± 0	6.8 ± 0			ns	8.2 ± 0	8.2 ± 0.1			ns	6.8 ± 0	6.8 ± 0			ns
	T5	1.6 ± 0.1	1.6 ± 0.1			ns	0.8 ± 0.1	0.8 ± 0.1			ns	7.1 ± 0.1	7.1 ± 0.1			ns	4.7 ± 0	4.7 ± 0			ns	4.5 ± 0.1	4.2 ± 0.1			ns
Merlot	T1	19.9 ± 0.2	21.1 ± 0.7	ns	1526 *	ns	1.7 ± 0.1	1.7 ± 0.2	ns	534 *	ns	18.1 ± 0.2	17.5 ± 1.1	ns	292.4 *	ns	1.5 ± 0.3	1.4 ± 0.2	ns	3263 *	ns	35 ± 2	37.8 ± 2.4	ns	21.56 *	ns
	T2	24.1 ± 0.6	26 ± 1			ns	n.d.	n.d.			ns	21.4 ± 0.5	23.4 ± 0.2			ns	1 ± 0.1	1.2 ± 0			ns	39.3 ± 0.3	38 ± 6.7			ns
	T3	26.8 ± 0.4	26.8 ± 0.2			ns	1.8 ± 0.3	1.9 ± 0.1			ns	20.3 ± 1.5	20.2 ± 0.4			ns	1.1 ± 0	1.1 ± 0			ns	37.7 ± 0.7	37.1 ± 5			ns
	T4	59.9 ± 0.5	59.3 ± 0.2			ns	4.8 ± 0.1	4.8 ± 0.1			ns	35.7 ± 0.1	35.4 ± 0.2			ns	12.7 ± 0.3	12.8 ± 0			ns	24.5 ± 0.1	23.6 ± 0.3			ns
	T5	28.7 ± 1.6	29.8 ± 0.5			ns	2.4 ± 0.3	2.7 ± 0.1			ns	42.5 ± 2.1	44.2 ± 2.3			ns	4.7 ± 0.2	4.9 ± 0.2			ns	24.2 ± 0.8	24.5 ± 0.1			ns
St Magdalener	T1	4.8 ± 0.3	4.7 ± 0.1	ns	404 *	ns	3.3 ± 0.2	3.3 ± 0.5	ns	21.9 *	ns	22.4 ± 1.9	20.3 ± 1.2	ns	474 *	ns	1.6 ± 0.2	1.5 ± 0	ns	2861 *	ns	30.2 ± 0.9	30.7 ± 1.1	ns	56.4 *	ns
	T2	5.4 ± 1	4.5 ± 0.2			ns	2.7 ± 0.2	1.8 ± 0.4			ns	23 ± 1.9	20.3 ± 1.1			ns	3.6 ± 0.2	3.3 ± 0			ns	40.4 ± 1.2	38.6 ± 1.2			ns
	T3	4.6 ± 0.2	4.3 ± 0.4			ns	2.4 ± 0.3	2.1 ± 0.2			ns	21.4 ± 0.3	20.2 ± 0.2			ns	1.5 ± 0.1	1.4 ± 0.3			ns	31.3 ± 0.1	28.6 ± 1.4			ns
	T4	15.2 ± 0.4	14.2 ± 0			ns	4.6 ± 0.9	4.2 ± 0.1			ns	39.4 ± 0.9	39.8 ± 0.4			ns	22.2 ± 0.8	21.1 ± 0			*	24.7 ± 5	27.3 ± 0.3			ns
	T5	6.2 ± 0.1	5.9 ± 0.6			ns	3.1 ± 0.1	3.2 ± 0.1			ns	46.9 ± 1	47.1 ± 0.6			ns	6.1 ± 0.1	6 ± 0.2			ns	22.1 ± 0.8	21.6 ± 0.2			ns
		**Caffeic Acid**					**Syringic Acid**					**(−)-Epicatechin**					***P*-coumaric Acid**					***Trans-*Resveratrol**				
	**Time**	**c**	**blend**	***F1***	***F2***	***F3***	**c**	**blend**	***F1***	***F2***	***F3***	**c**	**blend**	***F1***	***F2***	***F3***	**c**	**blend**	***F1***	***F2***	***F3***	**c**	**blend**	***F1***	***F2***	***F3***
Lagrein Red	T1	3.7 ± 0.1	3.6 ± 0	ns	1132 *	ns	7.7 ± 1	8.2 ± 0.4	ns	144 *	ns	26.2 ± 1.2	24.7 ± 0.5	ns	80 *	ns	6.1 ± 0.1	5.7 ± 0.1	8.2 *	4297 *	ns	3.4 ± 1.4	4 ± 0	ns	160.5 *	ns
	T2	3.8 ± 0	4.6 ± 0			ns	6.9 ± 0.1	6.5 ± 0.1			ns	28.2 ± 0.2	25 ± 0.3			ns	5.3 ± 0	4 ± 0.1			*	4.1 ± 0	4.2 ± 0.1			ns
	T3	4.1 ± 0.6	1.7 ± 0.1			*	6.1 ± 0.4	6.3 ± 0.5			ns	25.2 ± 1.8	20.5 ± 6.3			ns	6.1 ± 0.2	6.2 ± 0.5			ns	3.4 ± 0.5	3.6 ± 0			ns
	T4	27.4 ± 1.7	25.3 ± 0.2			*	3.9 ± 0	4.2 ± 0.1			ns	5.3 ± 0	4.7 ± 0.1			ns	24.7 ± 0.7	25.6 ± 0			ns	12.4 ± 0.8	11.8 ± 0.4			ns
	T5	2.1 ± 0.1	2.1 ± 0.4			ns	1.9 ± 0.3	1.8 ± 0			ns	10.7 ± 0.1	10.2 ± 0.4				3.7 ± 0	3.6 ± 0.1			ns	8.6 ± 0.2	8.7 ± 0.2			ns
Lagrein Rosè	T1	0.8 ± 0	0.8 ± 0	ns	41 *	ns	1.5 ± 0	1.5 ± 0.4	ns	24 *	ns	2.2 ± 0.3	1.1 ± 0.1	5.6 *	15 *	ns	2.8 ± 0.1	2.6 ± 0.2	6.8 *	1328 *	ns	2.4 ± 0.1	2.5 ± 0	ns	1899 *	ns
	T2	0.8 ± 0	0.7 ± 0			ns	1.3 ± 0.2	1.6 ± 0.4			ns	2.1 ± 0.6	2.8 ± 0.3			ns	3.5 ± 0.1	3.9 ± 0.2			*	2.5 ± 0	2.5 ± 0			ns
	T3	5.5 ± 1.7	4.1 ± 0.9			ns	1.3 ± 0.1	1.2 ± 0.2			ns	2.8 ± 0.9	1.5 ± 0			ns	2.9 ± 0	2.9 ± 0			ns	2.4 ± 0	2.5 ± 0			ns
	T4	2.4 ± 0.1	2.4 ± 0			ns	0.2 ± 0	0.2 ± 0			ns	1.9 ± 0	1.9 ± 0			ns	1.9 ± 0	1.8 ± 0			ns	0.4 ± 0	0.5 ± 0			ns
	T5	0 ± 0	0 ± 0			ns	0.9 ± 0	0.9 ± 0			ns	3.3 ± 0.1	3.7 ± 0.6			ns	0.1 ± 0	0.1 ± 0			ns	0.3 ± 0.1	0.2 ± 0			ns
Merlot	T1	1.9 ± 0.1	2.3 ± 0.1	ns	3435 *	ns	4.6 ± 1.1	3.9 ± 1.4	ns	30.3 *	ns	41.7 ± 0.2	42.7 ± 6.5	ns	274.1 *	ns	7.9 ± 0	7.6 ± 1	ns	282.7 *	ns	2.9 ± 0.7	3.5 ± 0.1	ns	143.3 *	ns
	T2	5.8 ± 0.2	6.2 ± 0.2			ns	3.8 ± 0.5	4.7 ± 0.8			ns	19.8 ± 1.6	21 ± 0.2			ns	8.6 ± 0.9	8.8 ± 0.8			ns	3.7 ± 0.1	3.4 ± 0.7			ns
	T3	1.7 ± 0	1.5 ± 0.1			ns	3.3 ± 0.4	3.2 ± 0.3			ns	41.2 ± 1.2	39.7 ± 5.1			ns	7.6 ± 0.4	7.5 ± 1.5			ns	3.6 ± 0	3.5 ± 0.1			ns
	T4	16 ± 0.1	16.1 ± 0.5			ns	2.3 ± 0.1	2.3 ± 0.1			ns	10.5 ± 0	10.1 ± 0.3			ns	19.4 ± 0.1	19.4 ± 0.2			ns	16 ± 0.1	15.4 ± 0.8			ns
	T5	0.7 ± 0.2	0.6 ± 0.2			ns	0.4 ± 0.1	0.5 ± 0.1			ns	16.7 ± 1	17 ± 0.4			ns	1.5 ± 0.1	1.6 ± 0			ns	8.1 ± 2.3	9.5 ± 0.4			ns
St Magdalener	T1	1.2 ± 0	1.2 ± 0.1	ns	517 *	ns	5.5 ± 0.4	5.4 ± 0.1	ns	319 *	ns	22.7 ± 1.1	21.1 ± 0.6	ns	101 *	ns	3.5 ± 0	3.7 ± 0.2	ns	705 *	ns	2.7 ± 0	2.7 ± 0	ns	22.8 *	ns
	T2	3.6 ± 0.5	2.5 ± 0.3			ns	4.3 ± 0.2	3.8 ± 0.5			ns	22.8 ± 0.4	20.1 ± 1			ns	6.4 ± 0.3	6 ± 0.4			ns	3.3 ± 0.1	3.4 ± 0.3			ns
	T3	1 ± 0	1 ± 0.1			ns	3.3 ± 0.3	3.2 ± 0.1			ns	18.3 ± 0.6	16.5 ± 0.2			ns	4.3 ± 0	3.8 ± 0			ns	2.6 ± 0	2.7 ± 0			ns
	T4	16.6 ± 1.8	17.6 ± 0.1			ns	0.8 ± 0.1	0.9 ± 0			ns	7.8 ± 2.8	9.3 ± 0			ns	15.4 ± 1	16.2 ± 0.2			ns	4.5 ± 1.2	3.5 ± 0.1			ns
	T5	0 ± 0	0 ± 0			ns	0.3 ± 0	0.3 ± 0			ns	12.1 ± 0.2	11.4 ± 0.6			ns	3.2 ± 0	3.2 ± 0.1			ns	1.4 ± 0	1.3 ± 0.4			ns

Legend: F1, F (interaction); F2, F (storage period); F3, F (stopper); blend, blend stopper; c, conventional stopper, as reported in Table 6. T1: samples at bottling; T2: after one month of storage at cellar temperature; T3: after three months of storage at cellar temperature; T4: after six months of storage at cellar temperature; T5: after 12 months of storage at cellar temperature. ns: not significant difference; *: significant difference (*p* < 0.05); n.d.: not detected.

**Table 2:** Anthocyanin concentrations (mg L^−1^) and significance of the type of stopper and storage time on their modifications. T1: samples at bottling; T2: after one month of storage at cellar temperature; T3: after three months of storage at cellar temperature; T4: after six months of storage at cellar temperature; T5: after 12 months of storage at cellar temperature.

	Glucoside						Acetyl-Glucoside					Cumaroyl-Glucoside				
	Time	c	Blend	*F1*	*F2*	*F3*	c	Blend	*F1*	*F2*	*F3*	c	Blend	*F1*	*F2*	*F3*
Lagrein red	T1	270.3 ± 6.2	266.2 ± 0.4	ns	1481 *	ns	87 ± 0.9	85.7 ± 0.1	ns	1694 *	ns	27.2 ± 0.6	27.6 ± 0.2	ns	1130 *	ns
	T2	253.3 ± 4	249.6 ± 3.3			ns	80.5 ± 0.6	79 ± 1.9			ns	26.1 ± 0.3	25.3 ± 0.5			ns
	T3	206.4 ± 0	193.1 ± 3.4			*	61.4 ± 0	58.9 ± 0.7			ns	20.7 ± 0.1	19.6 ± 0.4			ns
	T4	206 ± 0.4	198 ± 5.6			ns	69.8 ± 1	64.3 ± 2.7			*	21.1 ± 0	20.3 ± 0.8			ns
	T5	83.3 ± 5	80 ± 1.2			ns	17.1 ± 1.3	17.2 ± 0.4			ns	9.8 ± 0.2	10 ± 0.3			ns
Lagrein rosè	T1	28.2 ± 0.2	28.1 ± 0.2	ns	34170 *	ns	14.2 ± 0.1	14.3 ± 0	ns	20423 *	ns	7.4 ± 0	7.3 ± 0.1	ns	10927 *	ns
	T2	27.8 ± 0	27.8 ± 0.2			ns	14 ± 0.2	13.9 ± 0.1			ns	3.9 ± 0.1	4 ± 0.1			ns
	T3	25.7 ± 0	25.3 ± 0			ns	13 ± 0.1	13 ± 0			ns	3.8 ± 0	3.8 ± 0			ns
	T4	12.8 ± 0	12.6 ± 0.3			ns	4.1 ± 0.1	4 ± 0.1			ns	0.8 ± 0	0.7 ± 0.1			ns
	T5	5.8 ± 0	5.7 ± 0			ns	1 ± 0	1 ± 0			ns	0.4 ± 0	0.4 ± 0			ns
Merlot	T1	163.8 ± 1.4	159.7 ± 2.6	ns	3439 *	ns	46 ± 0.5	51.6 ± 0.9	15.5 *	1941 *	*	19.2 ± 0.3	18.9 ± 0.1	ns	979 *	ns
	T2	152.7 ± 0.7	149.9 ± 0			ns	42.8 ± 0.3	47.6 ± 0.5			*	18.6 ± 0.3	18.1 ± 0.1			ns
	T3	123.1 ± 0.2	121.5 ± 0.4			ns	35.7 ± 0.6	38.1 ± 0.9			*	15.6 ± 0.3	15.3 ± 0.1			ns
	T4	117.1 ± 0.7	114.4 ± 3.9			ns	30.4 ± 0.2	30.3 ± 1			ns	13.5 ± 0.1	13.1 ± 0.8			ns
	T5	48.4 ± 1.1	47.6 ± 0.4			ns	9.9 ± 0.1	9.6 ± 0.4			ns	7.2 ± 0.1	6.8 ± 0			ns
St Magdalener	T1	142.5 ± 1.7	142.7 ± 0.4	4.5 *	4767 *	ns	29.6 ± 0.1	28.9 ± 0.1	5.6 *	2971 *	ns	13.5 ± 0.2	13.4 ± 0	ns	1867 *	ns
	T2	138.6 ± 0	137.2 ± 0.1			ns	28.9 ± 0.1	28.6 ± 0.1			ns	13.4 ± 0.3	13.6 ± 0.2			ns
	T3	118.2 ± 1.1	116.2 ± 1.2			ns	24.6 ± 0.1	24.5 ± 0.4			ns	12.1 ± 0.1	12 ± 0.1			ns
	T4	113 ± 2.5	103.1 ± 2.9			*	16.2 ± 0.5	13.9 ± 0.7			*	10.6 ± 0.1	9.8 ± 0.7			ns
	T5	47.6 ± 0.3	43.1 ± 3.9			ns	5.2 ± 0	4.6 ± 0.4			ns	0.9 ± 0	1 ± 0.1			ns

Legend: F1, F (interaction); F2, F (storage period); F3, F (stopper); blend, blend stopper; c, conventional stopper, as reported in Table 6. Anthocyanin glucosides included delphinidin-3-*O*-glucoside, cyanidin-3-*O*-glucoside, petunidin-3-*O*-glucoside, peonidin-3-*O*-glucoside and malvidin-3-*O*-glucoside. Acetyl-glucosides included delphinidin-3-*O*-acetylglucoside, petunidin-3-*O*-acetylglucoside, peonidin-3-*O*-acetylglucoside, malvidin-3-*O*-acetylglucoside. Coumaroyl-glucosides included petunidin-3-*O*-coumaroylglucoside, peonidin-3-*O*-coumaroylglucoside and malvidin-3-*O*-coumaroylglucoside. ns: not significant difference; *: significant difference (*p* < 0.05).

**Table 3:** Dissolved oxygen (mg L^−1^).

	Time	Blend	c	Significant Difference (95% Confidence)
Lagrein Red	T1	0.40 ± 0.00	0.25 ± 0.07	
	T2	0.30 ± 0.00	0.20 ± 0.00	*
	T3	0.20 ± 0.00	0.25 ± 0.07	
	T4	0.20 ± 0.00	0.20 ± 0.14	
	T5	0.45 ± 0.07	0.50 ± 0.00	
Lagrein Rosé	T1	3.10 ± 0.00	1.40 ± 0.00	*
	T2	3.05 ± 0.07	2.20 ± 0.14	*
	T3	3.65 ± 0.07	2.45 ± 0.07	*
	T4	2.65 ± 0.07	1.90 ± 0.00	*
	T5	2.75 ± 0.07	2.90 ± 0.00	
Merlot	T1	1.70 ± 0.14	1.3 ± 0.28	
	T2	2.35 ± 0.21	1.80 ± 0.00	
	T3	2.75 ± 0.07	2.15 ± 0.07	*
	T4	1.25 ± 0.07	1.10 ± 0.00	
	T5	2.35 ± 0.07	2.55 ± 0.07	
St. Magdalener	T1	0.35 ± 0.07	0.15 ± 0.07	
	T2	0.30 ± 0.00	0.10 ± 0.00	*
	T3	0.40 ± 0.00	0.20 ± 0.00	*
	T4	0.10 ± 0.00	0.10 ± 0.00	
	T5	0.50 ± 0.14	0.45 ± 0.07	

Legend: Blend, blend stopper; c, conventional stopper, as reported in Table 6. * Significance level (*p* < 0.05) of difference between stoppers.

**Table 4:** List of volatile compounds (LRI, linear retention index).

No.	Esters	LRI [Ref.]
**2**	Ethyl butanoate	803 [28]
**3**	2-methylbutanoic acid, ethyl ester	846 [28]
**4**	3-methylbutanoic acid, ethyl ester	859 [29]
**6**	Isopentyl acetate	876 [30]
**7**	4-Ethylbenzoic acid, 2-butylester	-
**10**	Ethyl hexanoate	999 [30]
**11**	Hexyl acetate	1011 [30]
**14**	4-Methylbenzaldehyde	1076 [31]
**16**	4-Ethylbenzaldehyde	1163 [32]
**18**	Diethyl succinate	1179 [33]
**20**	Methyl salicylate	1192 [30]
**21**	Ethyl octanoate	1194 [34]
**22**	Benzenacetic acid ethyl ester	1243 [30]
**23**	2-Phenylethylacetate	1255 [30]
**24**	Ethyl decanoate	1392 [30]
**25**	Ethyl dodecanoate	1554 [30]
**26**	Ethyl hexadecanoate	1992 [35]
**No.**	**Alcohols**	**LRI [Ref.]**
**5**	1-Hexanol	865 [30]
**8**	1-Heptanol	969 [30]
**9**	1-Octen-3ol	980 [28]
**13**	2-Ethyl hexanol	1028 [36]
**15**	Octanol	1070 [30]
**17**	2-Phenylethyl alcohol	1112 [28]
**No.**	**Acids**	
**1**	Acetic acid	599 [28]
**19**	Octanoic acid	1180 [37]
**No.**	**Terpens**	
**12**	Limonene	1020 [30]

**Table 5:** Triangle test. ns: not significant; *: significant (*p* < 0.05).

	Lagrein Red	Lagrein Rosé	Merlot	St. Magdalener
T1 (Bottling time)	ns	ns	*	*
T2 (1 month)	ns	ns	ns	ns
T3 (3 months)	ns	ns	ns	ns
T4 (6 months)	ns	ns	*	ns
T5 (12 months)	ns	ns	ns	ns

**Table 6:** Types of closures used for the wine bottles.

Wines	Control	Conventional Stoppers
Lagrein red	Blend ^a^	One-piece natural cork
Lagrein Rosé	Blend	Technical cork 1+1
Merlot	Blend	Technical cork 1+1
St Magdalener	Blend	Agglomerated natural cork

^a^ Sanitized cork micro-granule blend of natural cork with polymers and without the addition of glue to the mix.

## Supplementary Materials

The following are available online. Tables S1: Tables of volatile compounds average peak areas in the studied wines.
