# Supplementary material for: Impact of Different Stoppers on the Composition of Red and Rosé Lagrein, Schiava (Vernatsch) and Merlot Wines Stored in Bottle
Source: Molecules. 2020 Sep 18;25(18):4276. doi: 10.3390/molecules25184276 (PMC7571222; doi:10.3390/molecules25184276)
Supplement: Supplementary file 1 [file molecules-25-04276-s001.pdf]

Table S1_A. Internal areas (average of two replicates) and standard deviations for volatiles compounds in Merlot wines.

		RELATIVE ABUNDANCES (INTERNAL AREAS) %																			
		T1				T2				T3				T4				T5			
Merlot	OTV (μg/mL)	c (Average)	c (st.dev.)	Blend (Average)	Blend (st.dev)	c (Average)	c (st.dev.)	Blend (Average)	Blend (st.dev)	c (Average)	c (st.dev.)	Blend (Average)	Blend (st.dev)	c (Average)	c (st.dev.)	Blend (Average)	Blend (st.dev)	c (Average)	c (st.dev.)	Blend (Average)	Blend (st.dev)
Acetic acid	200000[a]	0.00%	0.00%	0.00%	0.00%	0.00%	0.00%	0.00%	0.00%	5.16%	6.28%	0.88%	0.76%	3.80%	0.57%	3.20%	1.01%	0.47%	0.64%	0.75%	0.08%
Ethyl butanoate	20[a]	2.33%	0.03%	2.30%	0.19%	1.91%	0.21%	2.13%	0.05%	3.24%	0.17%	1.13%	0.45%	2.24%	0.01%	2.38%	0.78%	2.61%	0.47%	1.83%	0.06%
2-Methylbutanoic acid, ethyl ester	18[a]	0.31%	0.04%	0.31%	0.07%	0.39%	0.04%	0.51%	0.15%	0.33%	0.02%	0.22%	0.12%	0.61%	0.01%	0.52%	0.21%	0.58%	0.03%	0.64%	0.03%
3-Methylbutanoic acid, ethyl ester	3[a]	0.48%	0.05%	0.45%	0.08%	0.64%	0.09%	0.72%	0.07%	0.85%	0.03%	0.45%	0.27%	1.32%	0.11%	1.01%	0.37%	1.31%	0.04%	1.46%	0.10%
1-Hexanol	8000[a]	3.84%	0.21%	4.19%	0.12%	2.58%	0.59%	2.65%	0.62%	3.71%	0.57%	3.59%	0.43%	6.58%	0.17%	6.22%	2.39%	5.01%	0.92%	4.52%	0.66%
Isopentyl acetate	30[a]	3.05%	0.12%	3.38%	0.11%	2.85%	0.34%	3.07%	0.09%	5.21%	0.64%	9.68%	5.26%	5.10%	1.92%	4.07%	0.64%	3.23%	0.31%	8.26%	0.13%
4-Ethylbenzoic acid, 2-butyl ester		0.00%	0.00%	0.00%	0.00%	0.19%	0.12%	0.28%	0.07%	7.69%	1.47%	10.50%	2.41%	14.34%	5.23%	12.04%	3.58%	3.35%	0.28%	3.36%	0.10%
1-heptanol	1000[c]	0.00%	0.00%	0.00%	0.00%	0.00%	0.00%	0.00%	0.00%	0.38%	0.01%	0.30%	0.15%	0.00%	0.00%	0.00%	0.00%	0.35%	0.00%	0.32%	0.04%
1-octen-3ol		0.00%	0.00%	0.00%	0.00%	0.00%	0.00%	0.00%	0.00%	0.07%	0.02%	0.07%	0.01%	0.03%	0.04%	0.07%	0.06%	0.05%	0.06%	0.02%	0.00%
Ethyl hexanoate	14[a]	18.88%	1.09%	20.67%	0.99%	17.70%	0.07%	17.24%	1.44%	11.04%	0.85%	9.64%	1.85%	17.81%	1.29%	13.06%	6.31%	20.04%	1.45%	21.34%	0.80%
Hexyl acetate	670[c]	0.29%	0.01%	0.30%	0.03%	0.26%	0.02%	0.31%	0.05%	0.10%	0.01%	0.31%	0.29%	0.18%	0.03%	0.10%	0.06%	0.11%	0.03%	0.10%	0.04%
Limonene	200[c]	0.00%	0.14%	0.00%	0.27%	0.00%	0.01%	0.00%	0.01%	0.00%	0.05%	0.00%	0.07%	0.00%	0.00%	0.00%	0.06%	0.00%	0.04%	0.00%	0.03%
2-ethyl-1-hexanol	8000[c]	0.22%	0.00%	0.34%	0.00%	0.11%	0.00%	0.09%	0.00%	0.46%	0.00%	0.27%	0.00%	0.15%	0.00%	0.13%	0.00%	0.14%	0.00%	0.10%	0.00%
4-Methylbenzaldehyde		0.00%	0.00%	0.00%	0.00%	0.00%	0.00%	0.00%	0.00%	0.00%	0.00%	0.10%	0.00%	0.00%	0.00%	0.00%	0.00%	0.00%	0.00%	0.00%	0.00%
1-octanol	120[c]	0.61%	0.06%	0.35%	0.25%	0.51%	0.08%	0.45%	0.05%	1.55%	0.04%	0.61%	0.02%	1.15%	0.08%	1.02%	0.27%	0.91%	0.03%	0.84%	0.09%
4-Ethylbenzaldehyde		0.00%	0.00%	0.00%	0.00%	0.00%	0.00%	0.00%	0.00%	4.45%	1.31%	3.54%	0.05%	0.00%	0.00%	0.00%	0.00%	0.30%	0.10%	0.35%	0.04%
2-Phenylethyl alcohol	14000[a]	22.79%	6.05%	24.21%	0.37%	24.33%	1.26%	25.96%	1.45%	19.82%	4.46%	22.55%	4.47%	15.85%	1.10%	25.12%	15.31%	21.99%	1.56%	18.97%	1.75%
Diethyl succinate	1250[d]	8.95%	1.77%	9.90%	1.42%	11.87%	1.02%	12.27%	0.48%	16.68%	0.03%	19.58%	2.45%	20.07%	1.37%	23.05%	4.08%	22.03%	1.33%	18.84%	0.88%
Octanoic acid	10[b]	0.00%	0.00%	0.00%	0.00%	0.00%	0.00%	0.00%	0.00%	0.36%	0.18%	0.04%	0.05%	0.00%	0.00%	0.00%	0.00%	0.00%	0.00%	0.00%	0.00%
Methyl salicylate		0.00%	0.00%	0.00%	0.00%	0.04%	0.00%	0.03%	0.00%	5.19%	0.00%	3.57%	0.00%	0.04%	0.01%	0.04%	0.02%	0.04%	0.04%	0.07%	0.02%
Ethyl octanoate	2[c]	29.58%	6.26%	24.22%	0.37%	27.78%	2.95%	23.20%	2.10%	7.92%	0.44%	7.59%	0.86%	7.49%	0.23%	4.81%	2.56%	13.25%	0.37%	13.69%	2.24%
Benzenacetiacid ethyl ester		0.00%	0.00%	0.00%	0.00%	0.08%	0.00%	0.08%	0.01%	0.07%	0.01%	0.08%	0.00%	0.11%	0.01%	0.29%	0.20%	0.11%	0.05%	0.16%	0.03%
2-Phenylethylacetate	250[a]	0.57%	0.20%	0.77%	0.25%	0.71%	0.08%	0.80%	0.22%	0.75%	0.00%	1.51%	1.10%	1.33%	0.04%	1.38%	0.09%	0.51%	0.10%	0.56%	0.03%
Ethyl decanoate	200[a]	7.77%	0.70%	8.32%	0.59%	7.26%	0.62%	9.32%	1.18%	3.12%	0.25%	2.27%	0.53%	1.52%	0.16%	1.21%	0.38%	3.00%	0.51%	2.94%	0.34%

Ethyl dodecanoate	1500[c]	0.15%	0.06%	0.09%	0.13 %	0.46%	0.17%	0.51%	0.21 %	0.77%	0.21%	0.52%	0.03 %	0.19%	0.04%	0.19%	0.04 %	0.25%	0.14%	0.32%	0.06 %
Ethyl hexadecanoate		0.16%	0.06%	0.21%	0.02 %	0.32%	0.01%	0.36%	0.05 %	1.07%	0.21%	1.00%	0.03 %	0.11%	0.15%	0.08%	0.11 %	0.37%	0.27%	0.55%	0.03 %
Total abundance		100%		100%		100%		100%		100%		100%		100%		100%		100%		100%	

[a] Ferreira et al. J. Sci. Food Agric., 2000, Vol. 80(11), pp. 1659-1667.

[b] Peinado et al. Food Chem., 2006, Vol. 94, pp. 232-239.

[c] Jiang et al. Food Research International, 2013, Vol. 51, pp. 482–489

[d] Moyano et al. J Agric. Food Chem. 2002, Vol 50, pp. 7356-7361

Table S1_B. Internal areas (average of two replicates) and standard deviations for volatiles compounds in Lagrein wines.

RELATIVE ABUNDANCES (INTERNAL AREAS) %																			
T1				T2				T3				T4				T5			
c (Average)	c (st.dev.)	Blend (Average)	Blend (st.dev)	c (Average)	c (st.dev.)	Blend (Average)	Blend (st.dev)	c (Average)	c (st.dev.)	Blend (Average)	Blend (st.dev)	c (Average)	c (st.dev.)	Blend (Average)	Blend (st.dev)	c (Average)	c (st.dev.)	Blend (Average)	Blend (st.dev)
0.00%	0.00%	0.00%	0.00%	0.00%	0.00%	0.00%	0.00%	1.73%	0.06%	0.26%	0.36%	3.92%	2.46%	4.17%	1.27%	0.72%	0.32%	1.06%	0.89%
1.73%	0.31%	1.60%	0.17%	1.64%	0.52%	1.58%	0.11%	1.60%	0.58%	1.95%	0.01%	1.81%	0.93%	1.10%	0.00%	1.50%	0.09%	1.62%	0.27%
0.24%	0.03%	0.23%	0.05%	0.32%	0.08%	0.30%	0.02%	0.16%	0.00%	0.20%	0.08%	0.24%	0.07%	0.15%	0.02%	0.22%	0.07%	0.25%	0.04%
0.38%	0.08%	0.37%	0.07%	0.39%	0.05%	0.35%	0.10%	0.33%	0.02%	0.65%	0.15%	0.43%	0.09%	0.35%	0.08%	0.56%	0.28%	0.48%	0.03%
3.72%	0.26%	4.00%	0.33%	3.39%	0.32%	3.43%	0.19%	4.48%	0.76%	4.64%	0.14%	4.71%	0.09%	4.37%	0.11%	3.10%	0.16%	4.37%	0.15%
3.86%	0.47%	3.99%	0.43%	3.89%	0.96%	3.85%	0.03%	8.55%	1.26%	12.48%	0.67%	4.03%	1.31%	4.01%	0.10%	6.13%	3.15%	11.23%	0.07%
0.00%	0.00%	0.00%	0.00%	1.03%	0.93%	0.04%	0.05%	8.54%	1.24%	6.55%	0.17%	12.36%	3.54%	11.23%	0.52%	1.36%	0.10%	4.38%	0.17%
0.00%	0.00%	0.00%	0.00%	0.00%	0.00%	0.00%	0.00%	0.35%	0.08%	0.39%	0.00%	0.00%	0.00%	0.00%	0.00%	0.11%	0.02%	0.12%	0.09%
0.00%	0.00%	0.10%	0.14%	0.00%	0.00%	0.00%	0.00%	0.12%	0.03%	0.13%	0.00%	0.03%	0.01%	0.03%	0.00%	0.01%	0.00%	0.02%	0.00%
13.94%	1.04%	14.91%	0.17%	14.72%	2.77%	14.11%	0.09%	8.29%	1.48%	12.47%	0.66%	10.35%	1.92%	9.77%	0.35%	13.43%	3.61%	14.38%	0.08%
0.62%	0.03%	0.60%	0.01%	0.58%	0.05%	0.56%	0.02%	0.34%	0.08%	0.49%	0.01%	0.32%	0.08%	0.24%	0.00%	0.33%	0.10%	0.25%	0.03%
0.07%	0.02%	0.06%	0.01%	0.00%	0.00%	0.00%	0.00%	0.00%	0.00%	0.00%	0.00%	0.00%	0.00%	0.00%	0.00%	0.00%	0.00%	0.00%	0.00%
0.16%	0.07%	0.12%	0.08%	0.00%	0.00%	0.00%	0.00%	0.19%	0.05%	0.22%	0.02%	0.05%	0.01%	0.06%	0.01%	0.07%	0.01%	0.08%	0.02%
0.00%	0.00%	0.00%	0.00%	0.00%	0.00%	0.00%	0.00%	0.00%	0.00%	0.00%	0.00%	0.00%	0.00%	0.00%	0.00%	0.00%	0.00%	0.00%	0.00%
3.07%	3.52%	0.50%	0.05%	0.49%	0.33%	0.42%	0.01%	0.73%	0.18%	0.51%	0.11%	0.90%	0.13%	0.17%	0.00%	0.64%	0.00%	0.55%	0.10%
0.00%	0.00%	0.00%	0.00%	0.00%	0.00%	0.00%	0.00%	4.24%	0.79%	2.05%	0.44%	0.00%	0.00%	0.00%	0.00%	0.28%	0.05%	0.19%	0.09%
19.06%	5.33%	18.10%	4.42%	17.05%	2.19%	18.80%	0.34%	11.57%	1.02%	11.34%	0.62%	14.87%	3.16%	24.92%	0.15%	18.79%	2.68%	12.51%	3.46%
15.81%	1.34%	16.08%	3.04%	20.26%	0.65%	19.87%	2.54%	29.61%	2.33%	16.61%	0.77%	34.83%	1.04%	29.72%	2.53%	30.35%	2.83%	30.79%	3.51%
0.00%	0.00%	0.00%	0.00%	0.00%	0.00%	0.00%	0.00%	0.10%	0.07%	0.00%	0.52%	0.00%	0.00%	0.00%	0.00%	0.00%	0.00%	0.00%	0.00%
0.00%	9.62%	0.00%	6.42%	0.00%	3.98%	0.00%	3.56%	5.91%	2.27%	3.08%	0.24%	0.00%	1.90%	0.00%	0.01%	0.00%	3.96%	0.00%	0.73%
27.87%	0.00%	28.90%	0.00%	26.89%	0.02%	26.46%	0.03%	8.17%	0.06%	19.75%	0.05%	6.86%	0.07%	6.11%	0.03%	14.18%	0.01%	11.74%	0.02%

0.09%	0.08%	0.09%	0.02%	0.13%	0.00%	0.12%	0.03%	0.25%	0.23%	0.03%	0.03%	0.17%	0.07%	0.13%	0.04%	0.12%	0.01%	0.09%	0.02%
0.75%	3.70%	0.67%	2.44%	0.97%	0.85%	0.94%	0.14%	1.37%	0.61%	0.67%	0.03%	1.15%	1.17%	0.98%	0.18%	0.80%	1.45%	0.47%	0.16%
7.99%	0.15%	8.94%	0.14%	7.31%	0.13%	7.90%	0.34%	2.03%	0.01%	4.62%	0.03%	2.07%	0.18%	2.20%	0.07%	6.63%	0.00%	4.92%	0.02%
0.46%	0.03%	0.49%	0.00%	0.56%	0.11%	0.90%	0.14%	0.61%	0.35%	0.40%	0.01%	0.77%	0.03%	0.16%	0.01%	0.36%	0.02%	0.29%	0.12%
0.18%	0.00%	0.24%	0.00%	0.37%	0.00%	0.38%	0.00%	0.72%	0.00%	0.50%	0.00%	0.14%	0.00%	0.11%	0.00%	0.30%	0.00%	0.23%	0.00%
100.00%		100.00%		100.00%		100.00%		100.00%		100.00%		100.00%		100.00%		100.00%		100.00%	

Table S1_C. Internal areas (average of two replicates) and standard deviations for volatiles compounds in St.Magdalener wines.

	RELATIVE ABUNDANCES (INTERNAL AREAS) %																			
	T1				T2				T3				T4				T5			
St. Magdalener	c (Average)	c (st.dev.)	Blend (Average)	Blend (st.dev)	c (Average)	c (st.dev.)	Blend (Average)	Blend (st.dev)	c (Average)	c (st.dev.)	Blend (Average)	Blend (st.dev)	c (Average)	c (st.dev.)	Blend (Average)	Blend (st.dev)	c (Average)	c (st.dev.)	Blend (Average)	Blend (st.dev)
Acetic acid	0.00%	0.00%	0.00%	0.00%	0.00%	0.00%	0.00%	0.00%	0.67%	0.39%	0.76%	0.13%	1.69%	0.83%	1.45%	1.16%	0.27%	0.11%	0.40%	0.56%
Ethyl butanoate	1.44%	0.37%	1.43%	0.31%	1.00%	0.07%	0.99%	0.07%	1.33%	0.61%	1.11%	0.42%	1.57%	0.32%	1.21%	0.26%	1.03%	0.01%	1.35%	0.73%
2-Methylbutanoic acid, ethyl ester	0.24%	0.05%	0.22%	0.06%	0.20%	0.02%	0.21%	0.01%	0.34%	0.19%	0.22%	0.04%	0.31%	0.08%	0.31%	0.03%	0.27%	0.03%	0.41%	0.29%
3-Methylbutanoic acid, ethyl ester	0.40%	0.11%	0.37%	0.11%	0.34%	0.04%	0.34%	0.04%	0.71%	0.36%	0.46%	0.11%	0.56%	0.17%	0.54%	0.00%	0.55%	0.10%	0.87%	0.72%
1-Hexanol	4.01%	0.62%	4.09%	1.16%	2.87%	0.36%	3.20%	0.28%	4.53%	0.78%	4.56%	0.18%	6.52%	2.00%	6.40%	0.24%	3.78%	0.24%	4.70%	0.44%
Isopentyl acetate	6.47%	1.42%	6.08%	0.89%	5.67%	0.27%	5.67%	0.34%	14.19%	7.28%	18.24%	1.62%	7.68%	1.50%	8.13%	1.03%	16.46%	1.45%	12.16%	5.21%
4-Ethylbenzoic acid, 2 butylester	0.19%	0.11%	0.13%	0.06%	0.31%	0.07%	0.21%	0.13%	7.04%	0.96%	7.82%	1.80%	9.92%	1.95%	11.37%	1.61%	0.00%	0.00%	1.64%	2.28%
1-heptanol	0.28%	0.07%	0.28%	0.06%	0.00%	0.00%	0.00%	0.00%	0.37%	0.14%	0.37%	0.03%	0.00%	0.00%	0.00%	0.00%	0.00%	0.00%	0.17%	0.24%
1-octen-3ol	0.00%	0.00%	0.00%	0.00%	0.00%	0.00%	0.00%	0.00%	0.19%	0.09%	0.12%	0.01%	0.04%	0.01%	0.03%	0.00%	0.02%	0.00%	0.01%	0.01%
Ethyl hexanoate	13.98%	2.20%	13.68%	0.88%	12.33%	2.77%	13.47%	1.05%	13.29%	2.31%	11.46%	0.36%	12.75%	2.26%	11.77%	0.13%	12.71%	0.22%	16.89%	5.43%
Hexyl acetate	0.98%	0.08%	1.55%	0.10%	0.97%	0.09%	0.95%	0.04%	0.47%	0.38%	0.48%	0.01%	0.64%	0.10%	0.53%	0.02%	0.43%	0.06%	0.26%	0.25%
Limonene	0.12%	0.00%	0.13%	0.00%	0.00%	0.00%	0.00%	0.00%	0.00%	0.00%	0.00%	0.00%	0.00%	0.00%	0.00%	0.00%	0.00%	0.00%	0.00%	0.00%
2-ethyl hexanol	0.09%	0.01%	0.11%	0.02%	0.00%	0.00%	0.06%	0.00%	0.23%	0.06%	0.25%	0.03%	0.11%	0.03%	0.11%	0.00%	0.08%	0.01%	0.11%	0.02%
4-Methyl benzaldehyde	0.00%	0.00%	0.00%	0.00%	0.00%	0.00%	0.00%	0.00%	0.14%	0.04%	0.11%	0.01%	0.00%	0.00%	0.00%	0.00%	0.00%	0.00%	0.00%	0.00%
Octanol	0.00%	0.00%	0.15%	0.21%	0.00%	0.00%	0.30%	0.02%	0.58%	0.10%	0.51%	0.03%	1.06%	0.11%	1.00%	0.05%	0.71%	0.01%	0.84%	0.10%
4-Ethylbenzaldehyde	0.00%	0.00%	0.00%	0.00%	0.00%	0.00%	0.00%	0.00%	4.76%	1.69%	3.67%	0.25%	0.00%	0.00%	0.00%	0.00%	0.39%	0.15%	0.30%	0.02%

2-Phenyl ethyl alcohol	28.62%	3.59%	23.06%	5.73%	22.79%	2.96%	22.90%	2.99%	14.20%	6.18%	12.69%	2.82%	16.92%	5.57%	17.17%	0.12%	19.57%	2.44%	16.86%	4.73%
Diethyl succinate	15.21%	2.05%	12.46%	2.88%	13.02%	1.76%	13.62%	2.28%	11.82%	0.66%	11.43%	0.67%	25.27%	1.38%	24.98%	0.08%	26.34%	1.09%	24.05%	6.22%
Octanoic acid	0.00%	0.00%	0.00%	0.00%	0.22%	0.06%	0.11%	0.12%	0.00%	0.00%	0.00%	0.00%	0.00%	0.00%	0.00%	0.00%	0.00%	0.00%	0.00%	0.00%
Methyl salicylate	0.00%	0.00%	0.00%	0.00%	0.00%	0.00%	0.00%	0.00%	5.38%	1.32%	4.46%	0.48%	0.00%	0.00%	0.00%	0.00%	0.00%	0.00%	0.04%	0.05%
Ehtyl octanoate	19.41%	1.30%	24.68%	8.81%	28.32%	5.62%	25.31%	4.96%	13.79%	5.49%	15.44%	5.09%	8.83%	0.76%	8.18%	0.16%	11.65%	1.21%	12.55%	0.59%
Benzenacetiacid ethyl ester	0.06%	0.01%	0.06%	0.01%	0.05%	0.01%	0.06%	0.02%	0.03%	0.04%	0.00%	0.00%	0.19%	0.11%	0.22%	0.17%	0.10%	0.01%	0.14%	0.00%
2-Phenylethylacetate	2.31%	0.35%	2.19%	0.25%	1.79%	0.27%	2.00%	0.59%	1.57%	0.96%	0.86%	0.29%	3.56%	1.56%	3.96%	0.13%	1.20%	0.05%	1.38%	1.10%
Ethyl decanoate	5.71%	1.93%	8.68%	5.96%	9.00%	4.10%	9.46%	4.25%	2.78%	1.07%	3.72%	1.06%	1.68%	0.07%	1.94%	0.07%	4.09%	0.68%	3.96%	1.73%
Ethyl dodecanoate	0.31%	0.09%	0.46%	0.20%	0.74%	0.42%	0.80%	0.61%	0.63%	0.10%	0.40%	0.04%	0.14%	0.02%	0.13%	0.01%	0.18%	0.03%	0.33%	0.07%
Ethyl hexadecanoate	0.18%	0.00%	0.20%	0.00%	0.39%	0.00%	0.33%	0.00%	0.97%	0.00%	0.87%	0.00%	0.56%	0.00%	0.59%	0.00%	0.18%	0.00%	0.58%	0.00%
Total abundance	100.00%		100.00%		100.00%		100.00%		100.00%		100.00%		100.00%		100.00%		100.00%		100.00%	

Table S1_D. Internal areas (average of two replicates) and standard deviations for volatiles compounds in Rosè wines.

	RELATIVE ABUNDANCES (INTERNAL AREAS) %																			
	T1				T2				T3				T4				T5			
Lagrein Rosé	c (Average)	c (st.dev.)	Blend (Average)	Blend (st.dev)	c (Average)	c (st.dev.)	Blend (Average)	Blend (st.dev)	c (Average)	c (st.dev.)	Blend (Average)	Blend (st.dev)	c (Average)	c (st.dev.)	Blend (Average)	Blend (st.dev)	c (Average)	c (st.dev.)	Blend (Average)	Blend (st.dev)
Acetic acid	0.00%	0.00%	0.00%	0.00%	0.23%	0.32%	0.00%	0.00%	0.43%	0.40%	0.36%	0.47%	1.01%	0.10%	1.11%	0.45%	0.00%	0.00%	0.08%	0.12%
Ethyl butanoate	1.09%	0.43%	1.22%	0.47%	0.84%	0.27%	0.88%	0.38%	1.71%	0.67%	2.37%	0.01%	2.17%	1.71%	1.16%	0.31%	0.97%	0.09%	1.12%	0.15%
Butanoic acid, 2-methyl ethyl ester	0.03%	0.02%	0.03%	0.02%	0.04%	0.01%	0.04%	0.01%	0.06%	0.00%	0.07%	0.00%	0.06%	0.00%	0.04%	0.02%	0.05%	0.00%	0.04%	0.01%
Butanoic acid, 3-methyl ethyl ester	0.06%	0.03%	0.07%	0.04%	0.05%	0.02%	0.07%	0.01%	0.15%	0.02%	0.18%	0.01%	0.16%	0.02%	0.11%	0.05%	0.13%	0.01%	0.12%	0.02%
1-Hexanol	2.15%	1.00%	2.40%	0.93%	1.85%	0.66%	2.57%	0.16%	5.26%	0.84%	6.61%	0.30%	5.67%	0.22%	5.72%	0.30%	2.72%	0.23%	3.16%	0.01%
Isopentyl acetate	24.90%	8.18%	26.88%	8.03%	16.97%	1.46%	29.41%	1.08%	28.00%	6.12%	32.27%	1.63%	25.67%	1.44%	25.10%	2.00%	15.87%	2.79%	15.28%	0.06%
4-Ethylbenzoic acid, 2 butylester	0.00%	0.00%	0.00%	0.00%	0.00%	0.00%	0.00%	0.00%	4.45%	1.32%	4.04%	0.78%	2.98%	1.70%	3.12%	0.33%	0.00%	0.00%	0.00%	0.00%
1-heptanol	0.00%	0.00%	0.00%	0.00%	0.00%	0.00%	0.00%	0.00%	0.00%	0.00%	0.00%	0.00%	0.00%	0.00%	0.00%	0.00%	0.00%	0.00%	0.00%	0.00%
1-octen-3ol	0.00%	0.00%	0.00%	0.00%	0.00%	0.00%	0.00%	0.00%	0.00%	0.00%	0.00%	0.00%	0.00%	0.00%	0.00%	0.00%	0.00%	0.00%	0.00%	0.00%
Ethyl hexanoate	11.62%	3.93%	11.75%	3.91%	12.35%	0.59%	12.87%	0.07%	18.89%	0.33%	21.20%	0.36%	16.17%	1.08%	16.46%	0.61%	16.42%	0.98%	16.95%	0.39%
Hexyl acetate	7.80%	1.90%	7.39%	1.63%	7.03%	1.39%	7.16%	0.42%	4.46%	0.09%	4.87%	0.27%	4.67%	0.25%	4.32%	0.12%	4.19%	0.10%	3.74%	0.11%

Limonene	0.04%	0.03%	0.04%	0.03%	0.02%	0.02%	0.02%	0.01%	0.00%	0.00%	0.00%	0.00%	0.00%	0.00%	0.00%	0.00%	0.00%	0.00%	0.00%	0.00%
2-ethyl hexanol	0.02%	0.00%	0.03%	0.01%	0.01%	0.00%	0.01%	0.00%	0.10%	0.05%	0.18%	0.01%	0.03%	0.00%	0.03%	0.00%	0.00%	0.00%	0.00%	0.00%
4-Methyl benzaldehyde	0.00%	0.00%	0.00%	0.00%	0.00%	0.00%	0.00%	0.00%	0.11%	0.01%	0.11%	0.00%	0.00%	0.00%	0.00%	0.00%	0.00%	0.00%	0.00%	0.00%
Octanol	0.05%	0.02%	0.07%	0.00%	0.05%	0.02%	0.06%	0.01%	0.12%	0.01%	0.13%	0.01%	0.07%	0.00%	0.09%	0.03%	0.04%	0.01%	0.07%	0.00%
4-Ethylbenzaldehyde	0.00%	0.00%	0.00%	0.00%	0.00%	0.00%	0.00%	0.00%	7.53%	0.26%	5.48%	0.08%	0.00%	0.00%	0.00%	0.00%	0.13%	0.04%	0.25%	0.04%
2-Phenyl ethyl alcohol	4.21%	1.10%	4.64%	0.68%	3.86%	1.39%	4.04%	0.34%	4.57%	2.47%	3.45%	0.51%	8.19%	1.46%	8.72%	0.99%	2.20%	0.08%	2.47%	0.42%
Diethyl succinate	1.19%	0.48%	1.35%	0.28%	1.49%	0.91%	1.89%	0.38%	5.05%	1.03%	4.03%	0.43%	7.25%	0.46%	7.37%	0.54%	3.69%	0.07%	4.68%	0.55%
Octanoic acid	0.00%	0.00%	0.00%	0.00%	0.00%	0.00%	0.00%	0.00%	0.00%	0.00%	0.00%	0.00%	0.69%	0.51%	0.65%	0.01%	0.31%	0.02%	0.36%	0.04%
Methyl salicylate	0.00%	0.00%	0.00%	0.00%	0.00%	0.00%	0.00%	0.00%	6.18%	1.03%	3.31%	0.45%	0.00%	0.00%	0.00%	0.00%	0.00%	0.00%	0.00%	0.00%
Ehtyl octanoate	34.63%	10.65 %	33.23%	10.53 %	39.42%	2.02%	31.13%	2.58%	3.04%	0.21%	2.91%	0.06%	18.62%	0.19%	18.76%	0.47%	51.67%	3.94%	49.80%	1.37%
Benzenacetiacid ethyl ester	0.02%	0.01%	0.02%	0.00%	0.03%	0.02%	0.03%	0.00%	0.12%	0.09%	0.10%	0.02%	0.05%	0.01%	0.06%	0.01%	0.05%	0.01%	0.05%	0.00%
2-Phenylethylacetate	1.75%	0.62%	1.43%	0.15%	2.12%	1.38%	2.14%	0.29%	3.25%	0.45%	3.03%	0.04%	2.67%	0.19%	2.90%	0.28%	1.17%	0.04%	1.40%	0.10%
Ethyl decanoate	10.14%	7.72%	9.20%	6.32%	12.88%	6.56%	7.11%	0.48%	4.86%	0.52%	3.50%	0.10%	3.72%	0.11%	4.02%	0.33%	0.00%	0.00%	0.00%	0.00%
Ethyl dodecanoate	0.18%	0.01%	0.12%	0.08%	0.52%	0.31%	0.45%	0.01%	0.61%	0.15%	0.81%	0.34%	0.07%	0.01%	0.09%	0.00%	0.25%	0.01%	0.25%	0.00%
Ethyl hexadecanoate	0.11%	0.04%	0.10%	0.03%	0.21%	0.01%	0.13%	0.04%	1.04%	0.14%	0.99%	0.06%	0.09%	0.01%	0.17%	0.00%	0.15%	0.01%	0.19%	0.01%
Total abundance	100.00 %		100.00 %		100.00 %		100.00 %		100.00 %		100.00 %		100.00 %		100.00 %		100.00 %		100.00 %	
